# Association of *HLA-B* and *HLA-DRB1* polymorphisms with antithyroid drug-induced agranulocytosis in a Han population from northern China

**DOI:** 10.1038/s41598-017-12350-2

**Published:** 2017-09-20

**Authors:** Yayi He, Jie Zheng, Qian Zhang, Peng Hou, Feng Zhu, Jian Yang, Wenhao Li, Pu Chen, Shu Liu, Bao Zhang, Bingyin Shi

**Affiliations:** 1 Department of Endocrinology, The First Affiliated Hospital of Xi’an Jiaotong University, Xi’an, Shaanxi Province The People’s Republic of China; 2 Clinical Research Center, The First Affiliated Hospital of Xi’an Jiaotong University, Xi’an, Shaanxi Province The People’s Republic of China; 3 College of Medicine & Forensic, Health Science Center, Xi’an Jiaotong University, Xi’an, Shaanxi Province The People’s Republic of China; 4 Center for Translational Medicine, The First Affiliated Hospital of Xi’an Jiaotong University, Xi’an, Shaanxi Province The People’s Republic of China; 5 School of Public Health, Health Science Center, Xi’an Jiaotong University, Xi’an, Shaanxi Province The People’s Republic of China

## Abstract

Antithyroid drug (ATD)-induced agranulocytosis is associated with human leukocyte antigen (HLA) and nearby genes in Southeast Asian and European populations. The susceptibility of the Han population from northern China to ATD-induced agranulocytosis has not been reported. We evaluated the associations of genetic variants at the *HLA-B* and *HLA-DRB1* loci and 32 candidate single nucleotide polymorphisms (SNPs) with agranulocytosis in 29 patients with ATD-induced agranulocytosis and in 140 patients with Graves’ disease (GD) as controls. All subjects were of Han descent from northern China. HLA-B*27:05 (P = 1.10 × 10^−4^), HLA-B*38:02 (P = 2.41 × 10^−4^) and HLA-DRB1*08:03 (P = 1.57 × 10^−3^) were susceptibility HLA variants for ATD-induced agranulocytosis. All subjects carrying the HLA-B*27:05 allele had agranulocytosis. The odds ratios (ORs) comparing allele carriers to non-carriers were 66.24 (95% confidence interval (CI): 3.54–1239.66) for HLA-B*27:05, 7.525 (95% CI: 2.294–24.68) for HLA-B*38:02 and 4.316 (95% CI: 1.56–11.93) for HLA-DRB1*08:03. Two SNPs, rs2596487 (OR = 4.196, 95% CI = 2.086–8.441, P = 2.08 × 10^−5^) and rs2228391 (OR = 3.621, 95% CI = 1.596–8.217, P = 1.2 × 10^−3^), were independently associated with ATD-induced agranulocytosis. Subjects carrying the ‘A’ allele of rs1811197 or HLA-B*38:02 showed lower minimum granulocyte counts than non-carriers (P = 4.74 × 10^−4^ and P = 7.39 × 10^−4^, respectively). Our findings support the association between genetic variations of *HLA-B* and *HLA-DRB1* with ATD-induced agranulocytosis in a Han population from northern China.

## Introduction

Graves’ disease (GD) is the most common cause of hyperthyroidism, accounting for 60% to 80% of cases^1^. Antithyroid drugs (ATDs), including methimazole (MMI), carbimazole and propylthiouracil (PTU), have been widely used to treat patients with GD since their initial development in 1941^2^. These agents, however, cause various adverse reactions, such as liver dysfunction and skin rash. Agranulocytosis, which is defined as a granulocyte count of less than 0.5 × 10^9^/L after ATD administration, is the most serious adverse drug reaction observed during GD treatment. ATD-induced agranulocytosis may increase the risk of infections and sepsis in patients with GD and may even become a life-threatening event^2–4^.

The mechanism by which ATDs induce agranulocytosis is thought to be mediated by the immune system and associated with toxicity^2,3,5–7^. Antineutrophil cytoplasmic antibodies (ANCAs) might contribute to agranulocytosis since several target antigens, such as proteinase 3 and cathepsin G, are expressed on the surface of neutrophils^8–10^. In addition, ATDs also penetrate the bone marrow and exert direct toxic effects on bone marrow characteristics by inhibiting the generation and differentiation of pluripotent hematopoietic stem cells^11^. A certain genetic predisposition has been associated with drug-induced agranulocytosis^12–16^. The most extensively studied genes are genes encoding the major human leukocyte antigens (*HLAs*)^14,17–19^.

Genetic association studies have underscored the importance of the *HLA* genes within the major histocompatibility complex (*MHC*) locus as susceptibility loci for agranulocytosis caused by multiple drugs. Both HLA class I (*HLA-A, -B, -C, -E, -F*, and *-G*) and II (*HLA-DR, -DQ, -DM*, and *-DP*) molecules are involved in the presentation of antigens to T-cell receptors^20^. HLA-DRB1*08:03 was recently shown to contribute to the risk of ATD-induced agranulocytosis in Japanese and Taiwan Chinese populations^18,21^. The HLA-B*38:02 allele and single nucleotide polymorphisms (SNPs) in the HLA region were also associated with an increased risk of ATD-induced agranulocytosis in Taiwan and Hong Kong populations^21,22^. In White European populations, ATD-induced agranulocytosis is associated with the HLA-B*27:05 allele and with a set of SNPs located on chromosome 6^23^. Genetic differences have been observed between Han populations from southern and northern China^24,25^. We performed a case-control study to investigate the association between ATD-induced agranulocytosis and candidate genes and to investigate the genetic predictors of ATD-induced agranulocytosis in northern Chinese Han populations. Additionally, we explored the associations between genetic polymorphisms and the clinical severity of ATD-induced agranulocytosis.

## Results

### Demographic characteristics of the study cohort

All subjects were from the Han population in northern China. The general data for the 29 patients with ATD-induced agranulocytosis and the 140 controls with GD who were enrolled in this study are summarized in Supplementary Table S1. Among the 29 patients who developed agranulocytosis after ATD treatments, 3 were treated with PTU, and 26 were treated with MMI.

### HLA genotyping and association analysis

**Figure 1:**
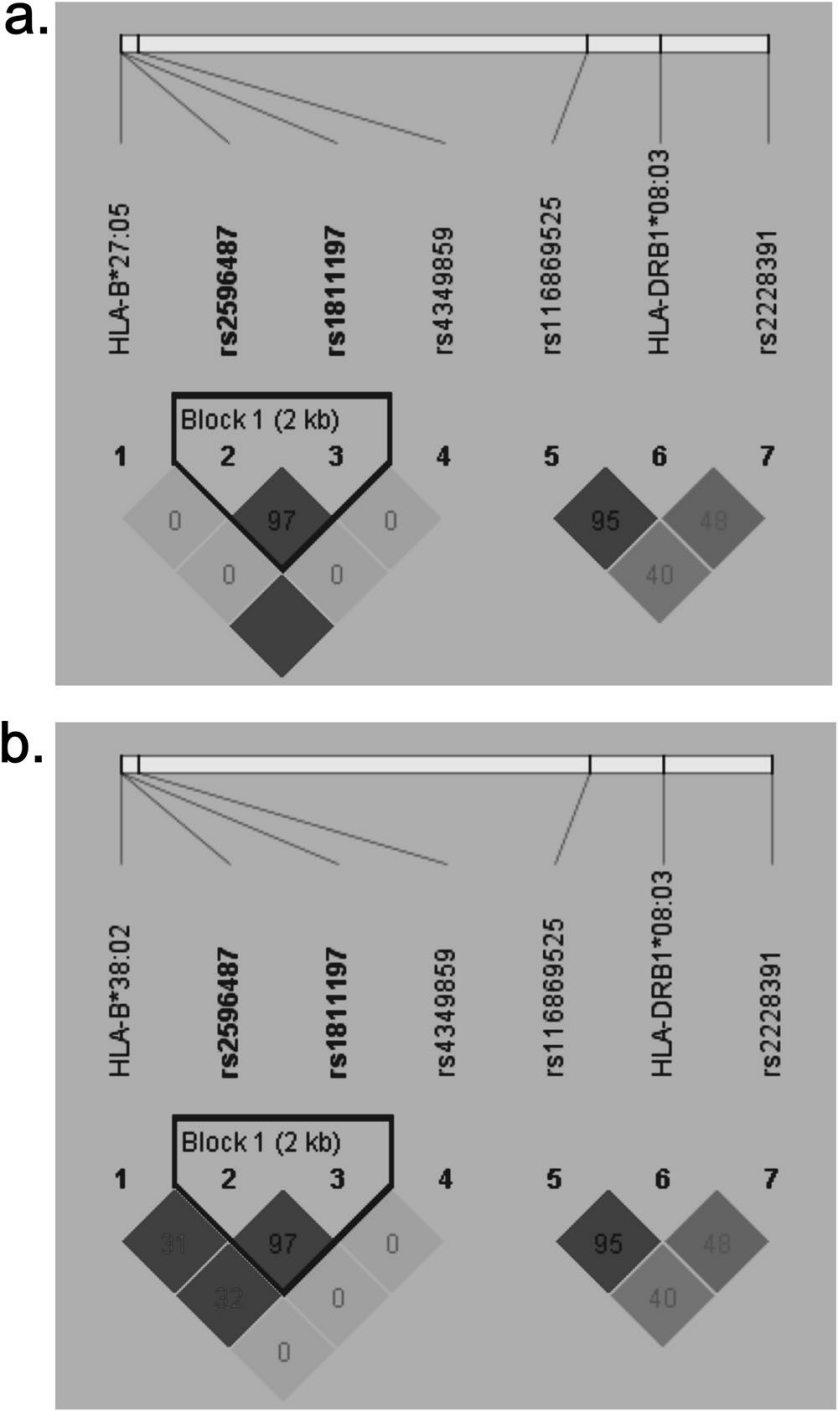
LD plots of (**a**) HLA-B*27:05, HLA-DRB1*08:03 and 5 SNPs; (**b**) HLA-B*38:02, HLA-DRB1*08:03 and 5 SNPs in 29 patients with ATD-induced agranulocytosis and 140 controls with GD. The numbers inside individual cells represent the r^2^ values (×100%) for the corresponding allele pairs.

Direct HLA genotyping was used to detect *HLA* variants (*HLA-B* and *HLA -DRB1*) in 29 patients with ATD-induced agranulocytosis and in 140 controls with GD. Genotyping data were missing for 2 patients and 5 controls in this study. Fifty-one *HLA-B* alleles and 35 *HLA-DRB1* alleles were detected in our population. Four *HLA-B* alleles (HLA-B*15:01, HLA-B*40:01, HLA-B*46:01 and HLA-B*51:01) and 6 *HLA-DRB1* alleles (HLA-DRB1*04:05, HLA-DRB1*12:01, HLA-DRB1*09:01, HLA-DRB1*15:01, HLA-DRB1*08:03 and HLA-DRB1*11:01) with allele frequencies greater than 5% were analyzed in the association study. Another 4 *HLA* alleles (HLA-B*38:02, HLA-B*27:05, HLA-DRB1*01:01 and HLA-DRB1*04:02) with frequencies less than 5% were also included in the analysis because of significant associations with drug-induced agranulocytosis that have been reported in the literatures^16,19,21–23,26^. In total, the association of 14 *HLA* alleles with ATD-induced agranulocytosis were analyzed. HLA-B*27:05 (P = 1.10 × 10^−4^), HLA-B*38:02 (P = 2.41 × 10^−4^) and HLA-DRB1*08:03(P = 1.57 × 10^−3^) were significantly associated with ATD-induced agranulocytosis after adjusting with Bonferroni’s correction (P < 3.57 × 10^−3^). The allele frequencies are shown in Table 1. HLA-B*27:05 was only detected in 18.5% of patients with agranulocytosis but not in the controls with GD, with an odds ratio (OR) of 66.24 (95% confidence interval, [CI] = 3.54–1239.66) for allele carriers compared with non-carriers. HLA-B*38:02 was present in 25.92% of patients with agranulocytosis but was present in only 4.4% of controls with GD with an odds ratio (OR) of 7.525 (95% confidence interval, CI = 2.294–24.68) for allele carriers compared with non-carriers. HLA-DRB1*08:03 was present in 29.6% of patients with agranulocytosis but was only present in 8.9% of controls with GD with an OR of 4.316 (95% CI = 1.56–11.93) for allele carriers compared with non-carriers. According to the linkage disequilibrium (LD) analysis, neither HLA-B*27:05 (Fig. 1a) nor HLA-B*38:02 (Fig. 1b) was in linkage disequilibrium with HLA-DRB1*08:03.

**Table 1:** Results of the association analysis using direct *HLA* typing.

*HLA* Allele	Position	Control MAF	Case MAF	OR (95% CI)	P	Control (Homozygous/ Heterozygous/ Non-carrier) (Carrier Percentage)	Case (Homozygous/ Heterozygous/ Non-carrier) (Carrier Percentage)	Allele carrier *vs*. Non-carrier ORs	
								OR (95% CI)	P(Add)
HLA-B*27:05	31355556	0/270 (0)	5/49 (0.093)	60.11 (3.272–1104.4)^¶^	1.1 × 10^−4^	0/0/135 (0)	0/5/22 (0.185)	66.24 (3.54–1239.66)^¶^	9.24 × 10^−5^
HLA-B*38:02	31355556	6/264 (0.022)	7/47 (0.130)	6.553 (2.11–20.36)	2.41 × 10^−4^	0/6/129 (0.044)	0/7/20 (0.259)	7.525 (2.294–24.68)	8.68 × 10^−4^
HLA-DRB1*08:03	32584303	13/257 (0.048)	9/45 (0.167)	3.954 (1.596–9.793)	1.57 × 10^−3^	1/11/123 (0.089)	1/7/19 (0.296)	4.316 (1.561–11.93)	2.8 × 10^−3^

MAF: minor allele frequency; CI: confidence interval; OR: odds ratio; Add: additive.

^¶^ORs and 95% CIs were estimated by adding 0.5 to all cells when a value of zero was present in one cell.

### Allelic and genotypic association analyses

Thirty-two SNPs were genotyped in the 29 patients with ATD-induced agranulocytosis and 140 controls with GD. All genotype frequencies were in Hardy-Weinberg equilibrium (HWE) in both patients and controls. The allele and genotype frequencies of all SNPs in both patients and controls are shown in Table 2. Among the 32 SNPs, 5 SNPs (rs2596487, rs1811197, rs2228391, rs116869525 and rs4349859) showed an association with ATD-induced agranulocytosis after adjusting with Bonferroni’s correction (P < 1.56 × 10^−3^). The linkage disequilibrium between the classical HLA alleles and the 5 SNPs is shown in Fig. 1. Rs4349859, which lies 41 kb centromeric of *HLA-B* and 5.4 kb telomeric of the MHC class I polypeptide-related sequence A *(MICA*) gene, exhibited high linkage disequilibrium with HLA-B*27:05. Rs116869525, which is located in the intergenic region between the *BTNL2* and *HLA-DRA* genes, exhibited high linkage disequilibrium with HLA-DRB1*08:03 (r^2^ = 0.95), and rs2596487 and rs1811197 were in high LD (r^2^ = 0.97) with each other.

**Table 2:** Allele and genotype frequency in the SNP association analysis.

Chr	SNPs (MAF)	Number (MAF)		P	OR (95%CI)	Genotype		P (Add)	OR (95%CI)
		Cases	Controls			Cases	Controls		
6	rs7768644 (A)	11/45 (0.196)	24/254 (0.086)	0.014	2.587 (1.185–5.649)	0/11/17	2/20/117	0.019	2.609 (1.168–5.826)
6	rs2517515 (T)	21/37 (0.362)	82/188 (0.304)	0.385	1.301 (0.718–2.359)	3/15/11	14/54/67	0.392	1.294 (0.717–2.334)
6	rs9263475 (G)	20/32 (0.385)	94/166 (0.362)	0.752	1.104 (0.598–2.038)	3/14/9	20/54/56	0.759	1.098 (0.605–1.991)
6	rs9262631 (A)	10/48 (0.172)	24/256 (0.086)	0.046	2.222 (0.999–4.943)	0/10/19	1/22/117	0.047	2.354 (1.013–5.472)
6	rs1265062 (T)	14/44 (0.241)	55/225 (0.196)	0.440	1.302 (0.666–2.543)	1/12/16	4/47/89	0.423	1.332 (0.660–2.687)
6	rs9263707 (T)	9/49 (0.155)	22/256 (0.079)	0.069	2.137 (0.929–4.919)	0/9/20	0/22/117	0.060	2.393 (0.964–5.940)
6	rs185386680 G)	5/45 (0.1)	8/266 (0.034)	0.019	3.694 (1.157–11.8)	0/5/20	0/8/129	0.024	4.031 (1.199–13.55)
6	rs34531986 (T)	15/43 (0.259)	41/235 (0.149)	0.041	1.999 (1.018–3.927)	0/15/14	5/31/102	0.048	1.998 (1.007–3.965)
6	rs2596487 (A)	17/41 (0.293)	25/253 (0.090)	**2.08 × 10** ^**−5**^	4.196 (2.086–8.441)	1/15/13	0/25/114	**4.38 × 10** ^**−5**^	5.620 (2.455–12.860)
6	rs13202464 (G)	10/46 (0.179)	25/253 (0.090)	0.048	2.200 (0.991–4.885)	2/6/20	1/23/115	0.064	2.088 (0.957–4.553)
6	rs2596449 (A)	8/46 (0.148)	27/237 (0.102)	0.326	1.527 (0.653–3.571)	1/6/20	3/21/108	0.361	1.449 (0.654–3.212)
6	rs2844505 (C)	9/49 (0.155)	31/247 (0.112)	0.350	1.463 (0.656–3.267)	1/7/21	3/25/111	0.375	1.416 (0.657–3.049)
6	rs805267 (A)	5/53 (0.086)	26/250 (0.094)	0.848	0.907 (0.333–2.471)	1/3/25	1/24/113	0.852	0.910 (0.341–2.435)
6	rs17201248 (T)	3/53 (0.054)	10/256 (0.038)	0.581	1.449 (0.386–5.444)	0/3/25	0/10/123	0.576	1.476 (0.379–5.751)
6	rs116869525 (T)	11/47 (0.190)	12/262 (0.044)	**7.062 × 10** ^**−5**^	5.110 (2.130–12.260)	1/9/19	1/10/126	**8.804 × 10** ^**−4**^	4.602 (1.872–11.31)
6	rs2228391 (C)	11/47 (0. 190)	17/263 (0.061)	**1.2 × 10** ^**−3**^	3.621 (1.596–8.217)	1/9/19	1/15/124	0.004	3.451 (1.494–7.968)
6	rs1143684 (C)	19/39 (0.328)	80/198 (0.288)	0.545	1.206 (0.657–2.212)	2/15/12	5/70/64	0.495	1.274 (0.636–2.552)
6	rs2530710 (T)	14/44 (0.241)	43/241 (0.154)	0.104	1.754 (0.885–3.474)	0/14/15	5/33/102	0.111	1.753 (0.880–3.495)
6	rs2517549 (A)	15/43 (0.259)	43/237 (0.154)	0.053	1.923 (0.982–3.763)	2/11/16	4/35/101	0.063	1.880 (0.965–3.665)
6	rs9263688 (G)	10/48 (0.172)	24/256 (0.086)	0.046	2.222 (1.999–4.943)	0/10/19	1/22/117	0.047	0.354 (1.013–5.472)
6	rs2770 (G)	15/43 (0.259)	81/197 (0.291)	0.616	0.848 (0.446–1.612)	3/9/17	20/41/78	0.656	0.878 (0.496–1.556)
6	rs2523608 (G)	29/27 (0.518)	135/145 (0.478)	0.590	1.171 (0.659–2.080)	9/11/8	52/31/57	0.660	1.111 (0.695–1.778)
6	rs2523605 (T)	10/48 (0.172)	57/223 (0.204)	0.588	0.815 (0.388–1.710)	1/8/20	9/39/92	0.610	0.833 (0.413–1.681)
6	rs1811197 (A)	16/42 (0.276)	25/255 (0.089)	**7.454 × 10** ^**−5**^	3.886 (1.916–7.882)	0/16/13	0/25/115	**6.409 × 10** ^**−5**^	5.662 (2.420–13.250)
6	rs4349859 (A)	5/53 (0.086)	0/278 (0)	**1.323 × 10** ^**−3**^	58.08(3.17–1066.99)	0/5/24	0/0/139	**1.13 × 10** ^**−4**^	^¶^62.63 (3.36–1169.12)
6	rs114291795 (G)	2/50 (0.038)	0/262 (0)	0.038	25.99(1.23–549.48)	0/2/24	0/0/131	0.027	^¶^26.84(1.25–576.29)
6	rs1799964 (C)	11/47 (0.190)	61/219 (0.218)	0.633	0.840 (0.411–1.718)	1/9/19	7/47/86	0.611	0.842 (0.413–1.716)
6	rs1799724 (T)	9/47 (0.161)	40/238 (0.144)	0.745	1.139 (0.518–2.505)	0/9/19	4/32/103	0.748	1.137 (0.521–2.480)
6	rs1800629 (A)	2/56 (0.034)	19/261 (0.068)	0.549	0.491 (0.111–2.167)	0/2/27	1/17/122	0.356	0.499 (0.114–2.181)
6	rs361525 (A)	0/56 (0)	15/265 (0.054)	0.146	0.15 (0.009–2.57)	0/0/28	0/15/125	0.078	^¶^0.142(0.008–2.444)
6	rs1800610 (A)	10/48 (0.172)	45/235 (0.161)	0.826	1.088 (0.513–2.308)	0/10/19	4/37/99	0.860	1.090 (0.509–2.332)
6	rs117968912 (T)	9/47 (0.161)	18/262 (0.064)	0.015	2.787 (1.181–6.575)	1/7/20	2/14/124	0.032	2.430 (1.078–5.478)

MAF: minor allele frequency; CI: confidence interval; OR: odds ratio; Add: additive. Significant P values are in bold.

^¶^ORs and 95% CIs were estimated by adding 0.5 to all cells when a value of zero was present in one cell.

**Figure 2:**
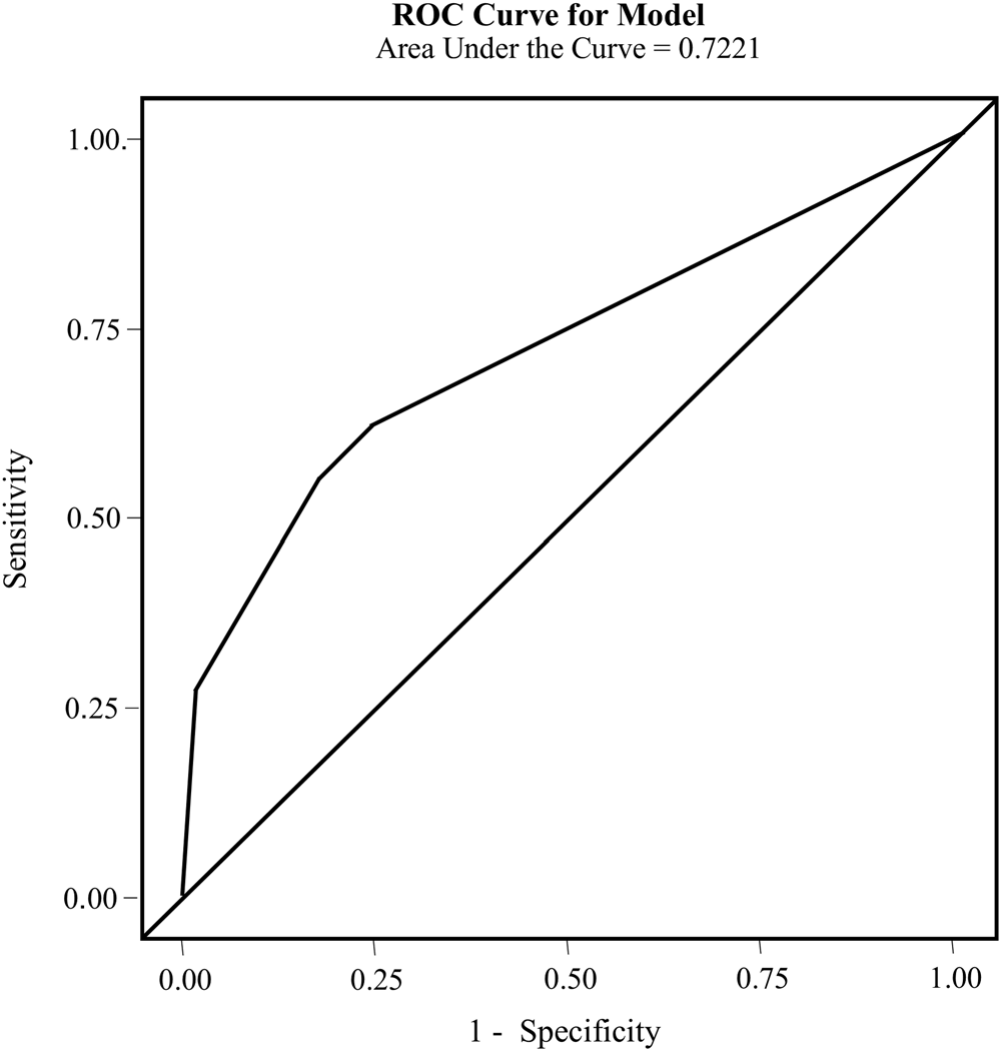
ROC curve used to discriminate patients with agranulocytosis (n = 29) from controls with GD (n = 140) on ATD-induced agranulocytosis. Two SNPs, rs116869525 and rs2596487 were included in the model to estimate the predicted probabilities. The sensitivity and specificity of the model were 0.621 and 0.759, respectively.

Logistic regression analyses were performed to investigate the contributions of the 5 significant SNPs to ATD-induced agranulocytosis. Rs4349859 and rs1811197 were not included in the analysis. Rs4349859 was not included in the regression model because no minor alleles (“A” allele of rs434985) were detected in the control patients with GD, and rs1811197 was not included in the model because of its high linkage disequilibrium with rs2596487. The final regression model revealed independent effects of rs2596487 (P = 0.0005) and rs116869525 (P = 0.0046) on ATD-induced agranulocytosis. In this model, the area under the receiver operating characteristic curve (AUC) was 0.722 (Fig. 2). The incidence rate of ATD-induced agranulocytosis was reported to be 0.001 to 0.005^2,4,27,28^. Assuming that the prevalence of ATD-induced agranulocytosis is 0.005 (one in 200 patients), we could theoretically reduce the incidence to 0.00190 (0.005–0.621 × 0.005)^22,23^ by genotyping for these two SNPs using an estimated sensitivity of 0.621. Approximately 322 patients (1/[0.005–0.00190]) should be screened by genotyping rs2596487 and rs116869525 to prevent one case of agranulocytosis.

### Stratification association study

We analyzed the patients and controls by separating males and females according to the results presented above to determine whether gender played a role in the association. Due to the limited number of the male patients in the present study, we only analyzed the female population. The associations of HLA-B*27:05, HLA-DRB1*08:03 and 5 SNPs (rs2596487, rs116869525, rs2228391, rs1811197 and rs4349859) with agranulocytosis were still significant in the female patients. When we considered medication type (MMI or PTU), the associations of two HLA alleles (HLA-B*38:02 and HLA-DRB1*08:03) and 2 SNPs (rs2596487 and rs1811197) with agranulocytosis were strengthened when patients with PTU-induced agranulocytosis were removed from the analysis (See Supplementary Tables S2 and S3).

### Associations of genotype with clinical parameters in patients with ATD-induced agranulocytosis

Associations of genetic variants and clinical parameters were analyzed in patients with ATD-induced agranulocytosis. The minimum granulocyte count was used to reveal the severity of agranulocytosis. As shown in Table 3, subjects carrying the GA genotype at rs1811197 showed lower minimum granulocyte counts (0.01 × 10^9^/L (0.00 × 10^9^/L–0.02 × 10^9^/L) *vs*. 0.19 × 10^9^/L (0.03 × 10^9^/L–0.43 × 10^9^/L), P = 4.74 × 10^−4^) than GG homozygotes. In addition, patients carrying the HLA-B*38:02 allele showed lower minimum granulocyte counts than HLA-B*38:02 non-carriers (0.00 × 10^9^/L (0.00 × 10^9^/L–0.01 × 10^9^/L) *vs*. 0.07 × 10^9^ /L (0.02 × 10^9^/L–0.42 × 10^9^/L), P = 7.39 × 10^−4^).

**Table 3:** Comparisons of the clinical parameters of patients with ATD-induced agranulocytosis among different genotypes.

SNPs or HLA variants	Genotypes/patients	Minimum granulocytes count (×10^9^)	Interval from ATD initiation to onset of agranulocytosis (days)	Onset age of agranulocytosis (years)
rs1811197 (n = 29)	GG(13)	0.19 (0.03–0.43)	25 (17–67.5)	44.46 ± 15.49
	AG(16)	0.01 (0.00–0.02)	58 (30–120)	41.69 ± 13.31
	P value	4.74 × 10^−4^^£^	0.118^£^	0.608*
HLA-B *38:02 (n = 27)	Heterozygous(7)	0.00 (0.00–0.01)	41.0 (20–84.25)	37.00 ± 11.46
	Non-carrier(20)	0.07 (0.02–0.42)	29.5 (19.5–86.25)	45.85 ± 14.55
	P value	7.39 × 10^−4^^£^	0.790^£^	0.159*

ATD: Antithyroid drug; Normally distributed variables (interval from ATDs initiation to onset of agranulocytosis, onset age of agranulocytosis) are reported as the means ± standard deviations (SD).

Minimum granulocyte counts are reported as medians with quartiles.

*A t-test was used to detect between-group differences for normally distributed variables.

^£^A non-parametric Mann-Whitney U test was used for non-normally distributed variables.

## Discussion

Agranulocytosis is the most dangerous side effect of ATD treatments, and the pathogenesis of ATD-induced agranulocytosis remains unclear. Recent genome-wide association studies (GWAS) have investigated the genetic predisposition of ATD-induced agranulocytosis in White European populations and Chinese populations in Hong Kong and Taiwan^21–23^. In this study, alleles of the classical *HLA* genes (HLA-B*27:05, HLA-B*38:02 and HLA-DRB1*08:03) and SNPs located in nearby genes (rs2596487 and rs1811197 located upstream of the *HLA-B* gene and rs2228391 on the transporter associated with antigen processing (*TAP*) gene) were associated with susceptibility of a northern Chinese Han population to ATD-induced agranulocytosis.

Substantial variations in *HLA* allele distributions have been observed across populations^29^. Even within the Chinese Han population, genetic differences have been observed among southern and northern populations^30^. Drug-induced agranulocytosis is associated with the *HLA* region but differs between populations. The HLA-B*38 allele was reported to be implicated in clozapine-induced agranulocytosis in Israeli Jewish patients with schizophrenia^14^ but not in other populations^12,16^. Recent studies have revealed a genetic contribution of the HLA-B*27:05 allele in predicting ATD-induced agranulocytosis in White European populations^23^, whereas HLA-B*38:02 and HLA-DRB1*08:03 are the genetic predictors of ATD-induced agranulocytosis in Chinese patients from Hong Kong^22^ and Taiwan^21^. In the present study, agranulocytosis occurred in all subjects carrying the HLA-B 27*05 allele, indicating a strong association of HLA-B*27:05 with the susceptibility of our population to ATD-induced agranulocytosis. The HLA-B*27:05 allele is common in European populations, with an allele frequency of 7.8%^23^. The allele frequencies of HLA-B*27:05 are 0% in Hong Kong and 0.1%–0.3% (extremely rare) in Taiwan populations, but relatively common in China Jiangsu (1.2%) and Yunnan Han (1.5%) populations^29^. Compared to HLA-B*27:05, the HLA-B*38:02 allele is common in southern Asian populations^29^, with an allele frequencies of 4%–7% in southern China^29,31^ and 1.9%–2.7% in northern China^29,32^. We speculated that the differences in *HLA* allele frequencies among populations might affect the ability to detect an association between certain *HLA* genes and ATD-induced agranulocytosis.

Five SNPs (rs4349859, rs2596487, rs1811197, rs2228391 and rs116869525) were associated with ATD-induced agranulocytosis in our population. First, rs4349859, which has been found to tag HLA-B*27 subtypes in patients of European descent with major ankylosing spondylitis^33^, was in high LD with HLA-B*27:05 in our population and showed a strong associations with ATD-induced agranulocytosis in a European population^23^ and agranulocytosis associated with levamisole-adulterated cocaine in Alberta and British Columbia populations^34^. Second, rs116869525, which was in high LD with HLA-DRB1*08:03 in both Chinese patients from Taiwan^21^ and our population, showed an association with ATD-induced agranulocytosis in both populations. Third, the top two SNPs, rs2596487 and rs 1811197, are located in upstream of the *HLA-B* genes. Rs2596487 was previously reported to be associated with susceptibility to ATD-induced agranulocytosis in European subjects^23^ and Chinese patients from Taiwan^21^. Rs1811197 was associated with ATD-induced agranulocytosis in European subjects^23^. Fourth, in addition to the *HLA* region, non-*HLA* genes also contribute to the susceptibility to agranulocytosis. Rs2228391, which is located in exon 4 of the *TAP2* gene, was previously shown to be associated with ATD-induced agranulocytosis in Chinese patients from Taiwan^21^. The *TAP2* gene encodes a subunit of TAP, which works with its binding protein, TAPBP, and participates in antigen presentation and processing.

In the previous GWAS from Hong Kong, HLA-B*38:02:01 was associated with CMZ/MMI-induced agranulocytosis but not with PTU-induced agranulocytosis^22^. Our findings are in line with the Hong Kong GWAS that the associations of HLA-B*38:02, HLA-DRB1*08:03, rs2596487 and rs1811197 with ATD-induced agranulocytosis become even stronger in patients with agranulocytosis caused only by MMI. Neither of the 2 PTU-induced agranulocytosis cases carried HLA-B*38:02 or HLA-DRB1*08:03. These findings suggest that the predisposing genetic factors for agranulocytosis induced by different antithyroid drugs might be different.

In this study, genetic variants were also helpful in predicting the severity of ATD-induced agranulocytosis. Patients carrying the “A” allele of SNP rs1811197 or the HLA-B*38:02 allele more easily develop severe agranulocytosis. However, the factors influencing the granulocyte count may be complicated and are not completely explained by genetics. Other factors, including delayed diagnosis and patient complications, are also involved in the severity of agranulocytosis^35^.

Our study and previous genetic studies provide a further explanation that genetic factors participate in the mechanism of ATD-induced agranulocytosis. Different major *HLA* genes or non-*HLA* genes may underlie the genetic predisposition of ATD-induced agranulocytosis but vary among populations.

The main limitation of our study is its single-center design. The number of subjects with ATD-induced agranulocytosis was relatively small, and the possibility of a population substructure cannot be completely excluded for any single genetic association study. However, despite this limitation, we identified a strong association between ATD-induced agranulocytosis and genetic polymorphisms. Further studies using larger samples with different regional backgrounds are needed to determine the validity of these associations, particularly within different ethnic populations.

In conclusion, HLA-B*27:05, HLA-B*38:02, HLA-DRB1 *08:03 and other SNPs within chromosome 6 are associated with the susceptibility of a Chinese Han population from northern China to ATD-induced agranulocytosis.

## Materials and Methods

### Study subjects

Subjects were diagnosed with GD based on clinical and biochemical hyperthyroidism, along with the presence of either thyroid exophthalmos or diffuse goiter and a significant titer of autoantibodies. ATDs, including MMI or PTU, were administered to all patients. Patients were diagnosed with ATD-induced agranulocytosis based on the criteria listed below. First, patients were confirmed to have a granulocyte count greater than or equal to 1.5 × 10^9^/L before they were administered ATDs. Second, the granulocyte count fell below 0.5 × 10^9^/L after ATD administration, and the patients recovered from agranulocytosis after the cessation of ATD treatment. Patients who had underlying hematological diseases (e.g., chronic neutropenia, myelodysplasia, aplastic anemia and pancytopenia) or systemic diseases (e.g., current systemic lupus erythematosus and current hepatocirrhosis) associated with neutropenia were excluded from the study. Subjects were also excluded from the study if they had a concomitant treatment known to affect leukocyte quantity (e.g., anticancer chemotherapy). The causality between ATDs and agranulocytosis was assessed using the World Health Organization – Uppsala Medical Centre (WHO-UMC) criteria with the causality term of ‘Certain’ in all 29 patients with ATD-induced agranulocytosis^36^. The Naranjo algorithm score was 7–8 (probable adverse drug reactions, ADR) in 20 patients because patients were not re-administered ATDs who were diagnosed with ATD-induced agranulocytosis^37^. The Naranjo algorithm score reached 9 (definite ADR) in the remaining 9 cases.

Because 80% of cases of ATD-induced agranulocytosis occur in the first 3 months^3,4,38,39^, the GD control group was composed of subjects with GD who had been treated with ATDs for at least 3 months and had not developed agranulocytosis upon entering the study. All control patients with GD were confirmed to have a normal granulocyte count during the subsequent treatment.

Between April 2013 and May 2016, 169 Chinese subjects, including 140 control patients with GD and 29 patients with ATD-induced agranulocytosis, were recruited from the inpatient and outpatient Endocrinology Departments of the First Affiliated Hospital of Xi’an Jiaotong University. Clinical data (including demographic information and medical history) were obtained from the control patients with GD in the outpatient department by reviewing their medical records. The medical records of patients with ATD-induced agranulocytosis were reviewed in detail. All data, including age of agranulocytosis onset, gender, interval days from ATD initiation to agranulocytosis onset, and biochemical test results (white blood cell and granulocyte counts) were obtained from the hospital’s computerized medical database.

This study was approved by the Medical ethics committee of the First Affiliated Hospital of Xi’an Jiaotong University (ethical approval no. KYLLSL-2013-107-01). All experimental procedures were performed according to standard guidelines and procedures approved by the above Ethics Committee. Informed consent was obtained from all participants.

### Genotyping

First, 28 SNPs located on chromosome 6 were considered candidates because of their previously reported involvement in agranulocytosis caused by either ATDs or other drugs^13,15,21–23^. Second, we examined marker SNPs in Haploview (v. 4.2) using the CHB (Han Chinese in Beijing, China) population and a minor allele frequency cut-off (MAF) of ≥5% (HapMap Data Release 27). Four marker SNPs within or near the *HLA-B* region were also screened as candidate loci because they are tag SNPs of the *HLA-B* gene or that region. Finally, 32 candidate SNPs were selected (Supplementary Table S4). Genomic DNA was isolated from peripheral blood samples using a genomic DNA kit (Tiangen Biotech Co., Ltd., China), according to the manufacturer’s protocol. SNP genotyping was performed using an iPLEX MassARRAY system (Sequenom, Inc., San Diego, CA, USA). Assay data were analyzed using Sequenom TYPER software (v. 3.4). The reliability of the subsequent statistical analysis was ensured by the high final genotype call rate for each SNP (greater than 98%) and the overall genotyping call rate (99.3%). Additionally, a random 5% of the samples were reanalyzed, and the results were 100% concordant.

### *HLA* typing and analysis

High resolution *HLA* sequence-based typing was performed. *HLA-B* polymorphisms on exon 2, exon 3 and exon 4, and *HLA-DRB1* polymorphisms on exon 2 were determined using the SeCore HLA Sequence-based Typing Kit (Capitalbio Corporation, China). The sequencing reaction for each exon was performed using a Big Dye terminator v3.1 cycle sequencing kit. Sequencing was performed on an automated ABI 3730 sequencer (Applied Biosystems, Foster City, CA, USA). The allele assignment was obtained by comparing the determined sequence with all combinations of known allele sequences in the IMGT/HLA database using the Assign-ATF sequence analysis software V1.0 (Conexio Genomics Pty Ltd., Australia). HLA association studies were performed using PLINK software version 1.9.

### Statistical analysis

The allele frequencies and genotype distributions for each SNP and HLA variant were descriptively summarized using PLINK software version 1.9 (Shaun Purcell, Christopher Chang, https://www.cog-genomics.org/plink2). The chi-square test or Fisher’s exact test was used to examine HWE for SNPs and HLA alleles. The association between genotype polymorphisms and the risk of ATD-induced agranulocytosis was estimated using P values, ORs, and 95% CIs. Haldane’s correction was performed when a value of zero was present in one cell. A P value cut-off of <1.56 × 10^−3^ was considered to indicate statistically significant after performing Bonferroni’s correction for the number of SNPs (n = 32), and a cut-off <3.57 × 10^−3^ was considered statistical significance after performing Bonferroni’s correction for the number of HLA alleles (n = 14). Pairwise LD statistics and haplotype frequencies were computed using Haploview 4.2 to construct haplotype blocks and to evaluate the association of haplotypes with ATD-induced agranulocytosis susceptibility^40^.

SPSS software (version 17.0; SPSS Inc., USA) was used to analyze differences in clinical parameters among different genotypes. A t-test was used to detect between-group differences for normally distributed variables (age of agranulocytosis onset), whereas the non-parametric Mann-Whitney U test was used for non-normally distributed variables (interval from ATD initiation to the onset of agranulocytosis and minimum granulocyte count). Normally distributed variables were reported as the means ± standard deviations (SD). The remaining continuous variables were reported as medians with quartiles due to their non-normal distributions.

## Electronic supplementary material

Supplementary tables

supplementary dataset

## References

[1] Weetman AP (2000). Graves’ Disease. New England Journal of Medicine..

[2] Cooper DS (2005). Antithyroid drugs. N Engl J Med..

[3] Watanabe N (2012). Antithyroid drug-induced hematopoietic damage: a retrospective cohort study of agranulocytosis and pancytopenia involving 50,385 patients with Graves’ disease. J Clin Endocrinol Metab..

[4] Nakamura H, Miyauchi A, Miyawaki N, Imagawa J (2013). Analysis of 754 cases of antithyroid drug-induced agranulocytosis over 30 years in Japan. J Clin Endocrinol Metab..

[5] Yang J (2013). The relationship between bone marrow characteristics and the clinical prognosis of antithyroid drug-induced agranulocytosis. Endocr J..

[6] Douer D, Eisenstein Z (1988). Methimazole-induced agranulocytosis: growth inhibition of myeloid progenitor cells by the patient’s serum. Eur J Haematol..

[7] Salama A, Northoff H, Burkhardt H, Mueller-Eckhardt C (1988). Carbimazole-induced immune haemolytic anaemia: role of drug-red blood cell complexes for immunization. Br J Haematol..

[8] Csernok E, Ernst M, Schmitt W, Bainton DF, Gross WL (1994). Activated neutrophils express proteinase 3 on their plasma membrane *in vitro* and *in vivo*. Clin Exp Immunol..

[9] Owen CA, Campbell MA, Boukedes SS, Campbell EJ (1995). Inducible binding of bioactive cathepsin G to the cell surface of neutrophils. A novel mechanism for mediating extracellular catalytic activity of cathepsin G. J Immunol..

[10] Akamizu T (2002). Drug-induced neutropenia associated with anti-neutrophil cytoplasmic antibodies (ANCA): possible involvement of complement in granulocyte cytotoxicity. Clin Exp Immunol..

[11] Waldhauser L, Uetrecht J (1991). Oxidation of propylthiouracil to reactive metabolites by activated neutrophils. Implications for agranulocytosis. Drug Metab Dispos..

[12] Corzo D (1995). The major histocompatibility complex region marked by HSP70-1 and HSP70-2 variants is associated with clozapine-induced agranulocytosis in two different ethnic groups. Blood..

[13] Turbay D (1997). Tumor necrosis factor constellation polymorphism and clozapine-induced agranulocytosis in two different ethnic groups. Blood..

[14] Valevski A (1998). HLA-B38 and clozapine-induced agranulocytosis in Israeli Jewish schizophrenic patients. Eur J Immunogenet..

[15] Ostrousky O (2003). NQO2 gene is associated with clozapine-induced agranulocytosis. Tissue Antigens..

[16] Opgen-Rhein C, Dettling M (2008). Clozapine-induced agranulocytosis and its genetic determinants. Pharmacogenomics..

[17] Hetherington S (2002). Genetic variations in HLA-B region and hypersensitivity reactions to abacavir. Lancet..

[18] Tamai H (1996). Association between the DRB1*08032 histocompatibility antigen and methimazole-induced agranulocytosis in Japanese patients with Graves disease. Ann Intern Med..

[19] Yunis JJ (1995). HLA associations in clozapine-induced agranulocytosis. Blood..

[20] Neefjes J, Jongsma ML, Paul P, Bakke O (2011). Towards a systems understanding of MHC class I and MHC class II antigen presentation. Nat Rev Immunol..

[21] Chen PL (2015). Genetic determinants of antithyroid drug-induced agranulocytosis by human leukocyte antigen genotyping and genome-wide association study. Nat Commun..

[22] Cheung CL (2016). HLA-B*38:02:01 predicts carbimazole/methimazole-induced agranulocytosis. Clin Pharmacol Ther..

[23] Hallberg P (2016). Genetic variants associated with antithyroid drug-induced agranulocytosis: a genome-wide association study in a European population. The Lancet Diabetes & Endocrinology..

[24] LL., C.-S., P., M. & A., P. The History and Geography of Human Genes. *Princeton: University Press* (1994).

[25] Wen B (2004). Genetic evidence supports demic diffusion of Han culture. Nature..

[26] Dettling M, Schaub RT, Mueller-Oerlinghausen B, Roots I, Cascorbi I (2001). Further evidence of human leukocyte antigen-encoded susceptibility to clozapine-induced agranulocytosis independent of ancestry. Pharmacogenetics..

[27] Tajiri J, Noguchi S (2004). Antithyroid drug-induced agranulocytosis: special reference to normal white blood cell count agranulocytosis. Thyroid..

[28] Takata K (2009). Methimazole-induced agranulocytosis in patients with Graves’ disease is more frequent with an initial dose of 30 mg daily than with 15 mg daily. Thyroid..

[29] Gonzalez-Galarza FF, Christmas S, Middleton D, Jones AR (2011). Allele frequency net: a database and online repository for immune gene frequencies in worldwide populations. Nucleic Acids Res..

[30] Pillai NE (2014). Predicting HLA alleles from high-resolution SNP data in three Southeast Asian populations. Hum Mol Genet..

[31] Trachtenberg E (2007). HLA class I (A, B, C) and class II (DRB1, DQA1, DQB1, DPB1) alleles and haplotypes in the Han from southern China. Tissue Antigens..

[32] Yang G (2006). HLA-A, -B, and -DRB1 polymorphism defined by sequence-based typing of the Han population in Northern China. Tissue Antigens..

[33] Evans DM (2011). Interaction between ERAP1 and HLA-B27 in ankylosing spondylitis implicates peptide handling in the mechanism for HLA-B27 in disease susceptibility. Nat Genet..

[34] Buxton JA (2015). Genetic determinants of cocaine-associated agranulocytosis. BMC Res Notes..

[35] He Y (2017). Emphasis on the early diagnosis of antithyroid drug-induced agranulocytosis: retrospective analysis over 16 years at one Chinese center. J Endocrinol Invest..

[36] Centre, T. U. M. The use of the WHO-UMC system for standardised case causality assessment. *Available**:* http://who-umc.org/Graphics/24734.pdf. (2013).

[37] Naranjo CA (1981). A method for estimating the probability of adverse drug reactions. Clin Pharmacol Ther..

[38] Yang, J. *et al*. Characteristics of antithyroid drug-induced agranulocytosis in patients with hyperthyroidism: A retrospective analysis of 114 cases in a single institution in China involving 9690 patients referred for radioiodine treatment over 15 years. *Thyroid* (2016). 10.1089/thy.2015.0439 26867063

[39] Kim HK (2015). Characteristics of Korean Patients with Antithyroid Drug-Induced Agranulocytosis: A Multicenter Study in Korea. Endocrinol Metab (Seoul)..

[40] Barrett JC, Fry B, Maller J, Daly MJ (2005). Haploview: analysis and visualization of LD and haplotype maps. Bioinformatics..

