# Supplementary material for: Association of HLA-B and HLA-DRB1 polymorphisms with antithyroid drug-induced agranulocytosis in a Han population from northern China
Source: Sci Rep. 2017 Sep 20;7:11950. doi: 10.1038/s41598-017-12350-2 (PMC5607267; doi:10.1038/s41598-017-12350-2)
Supplement: Supplementary file 1 — Supplementary tables [file 41598_2017_12350_MOESM1_ESM.pdf]

Association of *HLA-B* and *HLA-DRB1* polymorphisms with antithyroid drug-induced agranulocytosis in a Han population from northern China

Yayi He,[1] Jie Zheng,[2] Qian Zhang,[3] Peng Hou, [1] Feng zhu,[4] Jian Yang,[2] Wenhao Li,[5] Pu Chen, [1] Shu Liu,[1] Bao Zhang[*],[3] and Bingyin Shi[1*]

Supplementary Table S1 Demographic characteristics (gender and age) of the study cohort.

		Gender/ Age (Years)	
Group	N	Male	Female
ATD-induced agranulocytosis	29	3(33.33 ± 17.90)	26(44.04 ± 13.63)
GD controls	140	41(38.98 ± 12.87)	99(36.69 ± 12.93)

Data are reported as the means ± standard deviations (SD). Only ages are in brackets.

Supplementary Table S2 Allele and genotype frequencies after stratification of HLA associations by gender/treatment.

HLA Alleles	Gender/ Treatment	Case MAF	Control MAF	P	OR (95% CI)	Case (Homozygous /Heterozygous /Non-carrier) (Percentage)	Control (Homozygous /Heterozygous/ Non-carrier) (Percentage)	Homozygous+Heterozygous ORs	
								OR(95% CI)	P(Add)
*HLA-B*27:05*	Female	5/43 (0.1042)	0/190 (0)	**2.807×10^{-4}**	¶48.172 (2.61-887.60)	0/5/19 (0.208)	0/0/95 (0)	¶52.79 (2.81-993.64)	**2.33×10^{-4}**
	MMI	4/46 (0.08)	0/254 (0)	**6.601×10^{-4}**	¶49.26 (2.61-930.28)	0/4/21 (0.16)	0/0/127 (0)	¶57.37 (2.77-1027.15)	**5.92×10^{-4}**
*HLA-B*38:02*	Female	6/42 (0.125)	5/185 (0.02632)	3.620×10^{-3}	5.286 (1.54-18.14)	0/6/18 (0.250)	0/5/90 (0.053)	6 (1.651-21.8)	6.491×10^{-3}
	MMI	7/43 (0.14)	2/252 (0.0078)	**6.175×10^{-5}**	20.51 (4.123-102)	0/7/18 (0.28)	0/2/125 (0.0157)	24.31 (4.68-126.2)	**1.469×10^{-4}**
*HLA-DRB1*08:03*	Female	9/39 (0.1875)	10/180 (0.05263)	**2.068×10^{-3}**	4.154 (1.583-10.9)	1/8/15 (0.375)	1/9/85 (0.105)	3.611 (1.38-9.45)	**8.896×10^{-3}**
	MMI	9/41 (0.18)	12/242 (0.04724)	**7.15×10^{-4}**	4.427 (1.755-11.17)	1/7/17 (0.32)	1/10/116 (0.087)	3.855 (1.526-9.737)	4.319×10^{-3}

MMI: methimazole; MAF: minor allele frequency; CI: confidence interval; OR: odds ratio; Add: additive. Significant P values are in bold.

¶ORs and 95% CIs were estimated by adding 0.5 to all cells when a value of zero was present in one cell.

Supplementary Table S3 Allele and genotype frequencies after stratification of SNP associations by gender/treatment.

SNPs (MAF)	Gender/ Treatment	Number (MAF)		P	OR (95%CI)	Genotype		P (Add)	OR (95%CI)
		Cases	Controls			Cases	Controls		
rs116869525 (T)	Female	11/41 (0.212)	10/188 (0.055)	**1.94×10^{-4}**	5.044 (2.009-12.66)	1/9/16	1/8/90	0.0017	4.486 (1.771-11.88)
	MMI	10/42 (0.192)	11/245 (0.046)	**9.816×10^{-5}**	5.303 (2.120-13.26)	1/8/17	1/9/118	**1.3×10^{-3}**	4.637 (1.816-11.84)
rs2596487 (A)	Female	15/37 (0.289)	18/180 (0.091)	**1.8×10^{-4}**	4.054 (1.875-8.766)	0/15/11	0/18/81	**1×10^{-4}**	6.136 (2.419-15.56)
	MMI	16/36 (0.308)	20/240 (0.077)	**1.99×10^{-6}**	5.333 (2.532-11.23)	1/14/11	0/20/110	**1.14×10^{-5}**	7.387 (3.025-18.04)
rs2228391 (C)	Female	11/41 (0.212)	13/185 (0.066)	**1.5×10^{-3}**	3.818 (1.597-9.125)	1/9/16	0/13/86	0.0029	4.146 (1.624-10.59)
	MMI	10/42 (0.192)	16/246 (0.061)	0.0017	3.661 (1.557-8.609)	1/8/17	1/14/116	0.0054	3.431 (1.44-8.172)
rs1811197 (A)	Female	15/37	18/180	**1.8×10^{-4}**	4.054 (1.875-8.766)	0/15/11	0/18/81	**1.0×10^{-4}**	6.136 (2.419-15.56)

		(0.289)	(0.091)						
	MMI	15/37 (0.289)	20/242 (0.076)	**$9×10^{-6}$**	4.905 (2.309-10.42)	0/15/11	0/20/111	**$1.37×10^{-5}$**	7.568 (3.04-18.84)
rs4349859 (A)	Female	5/47 (0.096)	0/198 (0)	**$3.33×10^{-4}$**	¶45.97(2.50-845.79)	0/5/21	0/0/99	**$2.80×10^{-4}$**	¶50.91(2.71-955.61)
	MMI	4/48 (0.077)	0/260 (0)	**$6.99×10^{-4}$**	¶48.34(2.56-912.36)	0/4/22	0/0/130	**$6.30×10^{-4}$**	¶52.2(2.72-1003.13)

MMI: methimazole; MAF: minor allele frequency; CI: confidence interval; OR: odds ratio; Add: additive. Significant P values are in bold.

¶ORs and 95% CIs were estimated by adding 0.5 to all cells when a value of zero was present in one cell.

Supplementary Table S4 Summary of all candidate SNPs in this study.

SNP	Previous study	Populations		Samples		P value	CHR	Position	Nearby Gene	Frequencies (NCBI database)
		Ethnic group	Country	Case	Control					
rs116869525 (T)	Chen, PL2015	Southeast Asia	Taiwan	42 cases	927 GD controls	2.49×10^{-7}	6	32421366	*BTNL2 HLA-DRA*	T=0.0184/92
rs117968912 (T)	Chen, PL2015	Southeast Asia	Taiwan	42 cases	927 GD controls	2.47×10^{-7}	6	32713402	/	T=0.0256/128
rs1265062 (T)	Chen, PL2015	Southeast Asia	Taiwan	42 cases	927 GD controls	7.13×10^{-11}	6	31110228	/	T=0.3161/1583
rs140833037 (A)	Chen, PL2015	Southeast Asia	Taiwan	42 cases	927 GD controls	3.35×10^{-20}	6	31357279	*HLA-B*	T=0.0931/466
rs9262631 (A)	Chen, PL2015	Southeast Asia	Taiwan	42 cases	927 GD controls	7.34×10^{-19}	6	31056824	/	A=0.0667/334
rs17201248 (T)	Chen, PL2015	Southeast Asia	Taiwan	42 cases	927 GD controls	4.01×10^{-7}	6	31835353	*SNORD48*	T=0.0379/190
rs7768644 (A)	Chen, PL2015	Southeast Asia	Taiwan	42 cases	927 GD controls	5.86×10^{-13}	6	31034324	*MUC22*	A=0.1032/517
rs805267 (A)	Chen, PL2015	Southeast Asia	Taiwan	42 cases	927 GD controls	1.18×10^{-6}	6	31671980	*LY6G5B*	A=0.1190/596
rs9263475 (G)	Chen, PL2015	Southeast Asia	Taiwan	42 cases	927 GD controls	1.47×10^{-7}	6	31082571	/	G=0.3437/1721
rs9263688 (G)	Chen, PL2015	Southeast Asia	Taiwan	42 cases	927 GD controls	4.69×10^{-21}	6	31124190	*PSORS1C 1*	G=0.0563/282
rs9263707 (T)	Chen, PL2015	Southeast Asia	Taiwan	42 cases	927 GD controls	5.73×10^{-21}	6	31127469	*PSORS1C 1*	T=0.0565/283

rs2228391 (C)	Chen, PL2015	Southeast Asia	Taiwan	42 cases	927 GD controls	5.57×10^{-7}	6	32829996	*TAP2*	C=0.0302/151
rs2517515 (T)	Chen, PL2015	Southeast Asia	Taiwan	42 cases	927 GD controls	1.52×10^{-6}	6	31061481	/	T=0.2726/1365
rs2517549 (A)	Chen, PL2015	Southeast Asia	Taiwan	42 cases	927 GD controls	1.47×10^{-10}	6	31040821	/	A=0.2959/1482
rs2530710 (T)	Chen, PL2015	Southeast Asia	Taiwan	42 cases	927 GD controls	1.00×10^{-12}	6	30972610	/	T=0.1386/694
rs2596449 (A)	Chen, PL2015	Southeast Asia	Taiwan	42 cases	927 GD controls	6.22×10^{-14}	6	31470613	*HCG26*	A=0.1725/864
rs2844505 (C)	Chen, PL2015	Southeast Asia	Taiwan	42 cases	927 GD controls	6.50×10^{-14}	6	31471286	*HCG26*	C=0.1725/864
rs34531986 (T)	Chen, PL2015	Southeast Asia	Taiwan	42 cases	927 GD controls	5.79×10^{-18}	6	31347810	/	T=0.1392/697
rs1811197 (A)	Hallberg, 2016	European	6 countries	39 cases	5,161 population controls	9.18×10^{-7}	6	31359883	*HLA-B, LOC1053 75016*	A=0.0976/489
rs114291795 (G)	Hallberg, 2016	European	6 countries	38 cases	5,144 population controls.	4.10×10^{-8}	6	31409863	*MICA*	G=0.0130/65
rs4349859 (A)	Hallberg, 2016	European	6 countries	39 cases	5,400 population controls.	3.86×10^{-15}	6	31398010	*LOC1019 29072, MICA*	A=0.0136/68
rs185386680 (G)	Cheung, 2016	Asia	Southern Chinese descent	20 cases	775 healthy controls	9.6×10^{-7}	6	31207153	/	G=0.0100/50
rs1800629 (A)	Turbay D, 1997	The genetic variants of the TNF-α loci are associated with susceptibility to					6	31575254	*TNF*	A=0.0903/452

rs361525 (A)	Turbay D, 1997	clozapine induced agranulocytosis					6	31575324	*TNF*	A=0.0609/305
rs1799724 (T)	Turbay D,1997						6	31574705	*TNF*	T=0.0990/496
rs1799964 (C)	Turbay D,1997						6	31574531	*TNF*	C=0.2190/1097
rs1800610 (A)	Turbay D,1997						6	31576050	*TNF*	A=0.1004/503
rs1143684 (C)	Ostrousky O, 2003	The NQO2 gene is associated with clozapine-induced agranulocytosis					6	3010156	*NQO2*	C=0.2025/1014
rs13202464 (G)	Tag SNP	/	/	/	/	/	6	31376806	/	G=0.0713/357
rs2523605 (T)	Tag SNP	/	/	/	/	/	6	31355632	*HLA-B*	T=0.1274/638
rs2523608 (G)	Tag SNP	/	/	/	/	/	6	31354782	*HLA-B, MIR6891*	G=0.3972/1989
rs2770 (G)	Tag SNP	/	/	/	/	/		31354030	*HLA-B*	G=0.4067/2037

CHR: chromosome; SNP: single-nucleotide polymorphism; GD: Graves' disease; CA: Clozapine-induced agranulocytosis. The physical positions were annotated according to NCBI genome version of GRCh38.p7.

Supplementary references:

1. Chen, P. L. *et al.* Genetic determinants of antithyroid drug-induced agranulocytosis by human leukocyte antigen genotyping and genome-wide association study. *Nat Commun*. **6(**7),633 (2015).
2. Hallberg, P. *et al.* Genetic variants associated with antithyroid drug-induced agranulocytosis: a genome-wide association study in a European population. *The Lancet Diabetes & Endocrinology*. **4(6),** 507-516 (2016).
3. Cheung, C. L. *et al.* HLA-B*38:02:01 predicts carbimazole/methimazole-induced agranulocytosis. *Clin Pharmacol Ther*. **99(5),** 555-561 (2016).
4. Turbay, D. *et al.* Tumor necrosis factor constellation polymorphism and clozapine-induced agranulocytosis in two different ethnic groups. *Blood*. **89(11),** 4167-4174 (1997).
5. Ostrousky, O. *et al.* NQO2 gene is associated with clozapine-induced agranulocytosis. *Tissue Antigens*. **62(6),** 483-491 (2003).
